# A specialized role played by a redox cofactor

**DOI:** 10.1016/j.engmic.2022.100010

**Published:** 2022-01-10

**Authors:** Shengling Xie, Lihan Zhang

**Affiliations:** a Department of Chemistry, Zhejiang University, Hangzhou 310058, Zhejiang Province, China; b Key Laboratory of Precise Synthesis of Functional Molecules of Zhejiang Province, School of Science, Westlake University, 18 Shilongshan Road, Hangzhou 310024, Zhejiang Province, China; c Institute of Natural Sciences, Westlake Institute for Advanced Study, 18 Shilongshan Road, Hangzhou 310024, Zhejiang Province, China

**Keywords:** Biosynthesis, Nicotinamide adenine dinucleotide, Pyridoxal phosphate-dependent enzyme, Cycloaddition

## Abstract

The redox cofactor β-nicotinamide adenine dinucleotide (NAD) has been revealed to serve as a building block for the biosynthesis of an alkaloid natural product, altemicidin. The biosynthetic pathway investigation identified a unique pyridoxal pyrophosphate (PLP)-dependent enzyme that utilizes NAD and *S*-adenosylmethionine (SAM) to build the bicyclic alkaloid scaffold, opening the door for biosynthetic studies of NAD-derived natural products.

Natural products, or specialized metabolites, exhibit vast structural diversity and play important roles as antibiotics, signaling molecules, and siderophores in the natural environment. The unique structure of each molecule allows them to interact with different targets, thereby making natural products a primary source for drug discovery (Newman D. J. and Cragg G. M., 2020). Contrary to their structural diversity, however, the living organisms usually only use a limited set of molecules—sugars, amino acids, and their metabolites—for the biosynthesis of natural products. With the advances in biosynthetic research, we can now classify the origin of natural products into several categories based on their common structural features, *e.g.*, polyketides biosynthesized from C_2_-acetate units (Hertweck C., 2009); peptides from amino acids (Sussmuth R. D. and Mainz A., 2017); terpenes from C_5_-isoprene units etc. (Christianson D. W., 2008; Helfrich E. J. N. *et al*, 2019), which helps us understand the biosynthetic origin and the enzymatic transformation during the biogenesis of these natura products. Nevertheless, there are still many natural products that have not been biosynthetically classified, and altemicidin (Takahashi A. *et al*, 1989a; Takahashi A. *et al*, 1989b), an anticancer compound from soil streptomyces bearing a unique nitrogen-containing 6,5-bicyclic scaffold, is such a molecule with no apparent biosynthetic classification.

How is altemicidin generated in the microbe? Recent report by Abe, Awakawa, and co-workers revealed an unexpected biosynthetic pathway of altemicidin that begins with the condensation of two cofactors, β-nicotinamide adenine dinucleotide (NAD) and *S*-adenosylmethionine (SAM), catalyzed by a pyridoxal pyrophosphate (PLP)-dependent enzyme SbzP (Fig.1) (Barra L. *et al*, 2021). Previously, the authors had identified its biosynthetic gene cluster by resistance-guided genome mining and characterized the function of late-stage modification enzymes (Hu Z. *et al*, 2019). To further investigate the bicyclic core scaffold biosynthesis, the authors screened the remaining enzymes by heterologous expression, and revealed that SbzP is responsible for the synthesis of a nucleoside intermediate of altemicidin bearing the 6,5-bicyclic ring structure. Further isotope labeling experiments and in vitro enzyme assays demonstrated that SbzP exclusively utilizes NAD and SAM as substrates. Notably, despite being an essential molecule identified in the 1930s as the cofactor of glucose-6-phosphate dehydrogenase (Fang E. F. *et al*, 2017), the structural incorporation of NAD in natural products biosynthesis is unprecedented. Moreover, the SbzP-catalyzed cycloaddition proceeds in an unusual [3+2] manner, contrary to the more commonly observed [4+2] cycloaddition (Jeon B. S. *et al*, 2017). Subsequently, the team reconstituted the complete downstream biosynthetic pathway of altemicidin by systematic analysis of metabolites in gene knockout mutants.

**Fig. 1:**
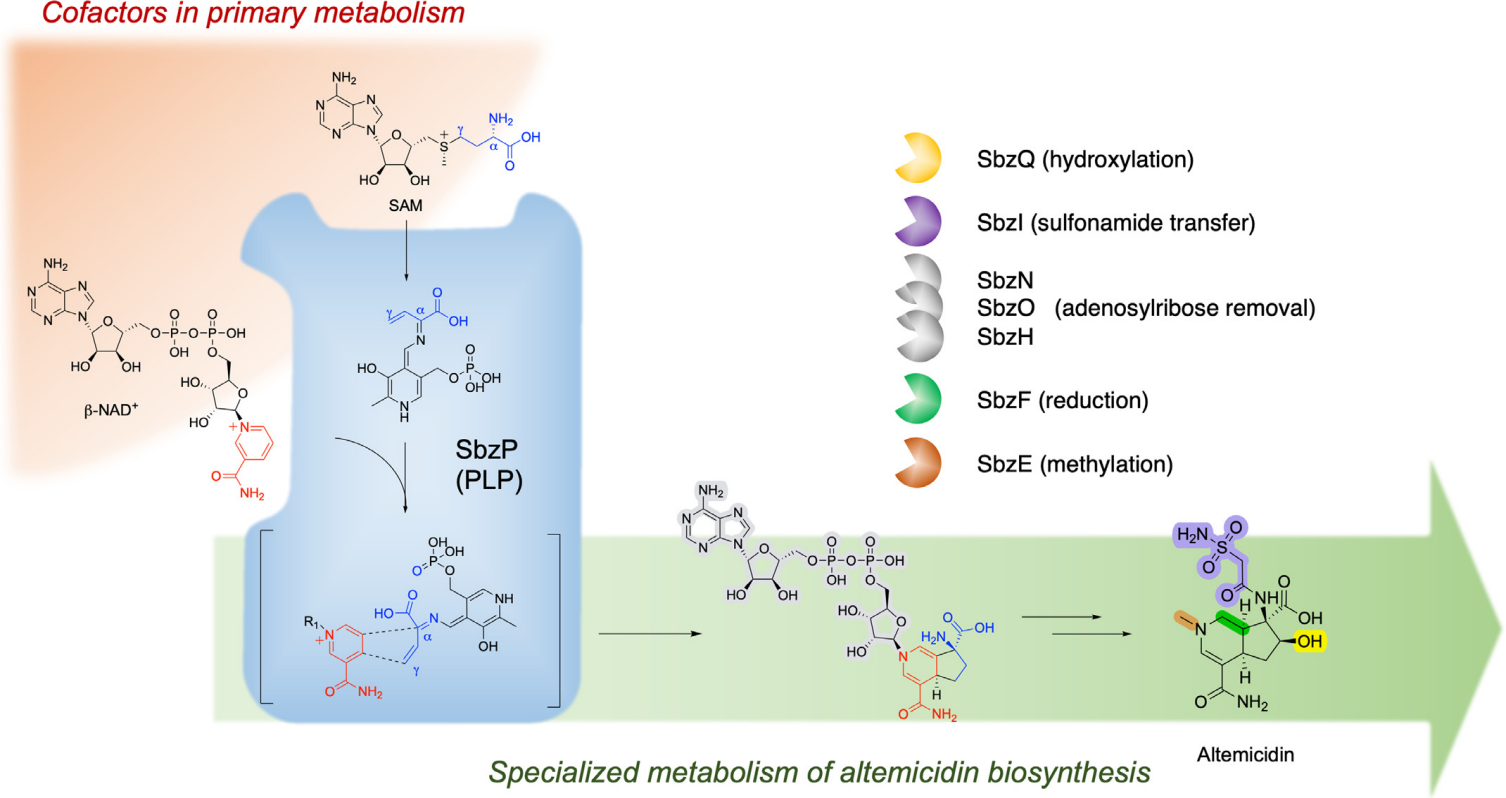
The discovered β-NAD-utilizing *sbz* biosynthetic pathway. The PLP dependent enzyme SbzP (middle) utilizes cofactors NAD^+^ and SAM to construct the altemicidin scaffold, bridging the primary metabolism and the specialized metabolism of altemicidin biosynthesis in bacteria.

The authors then investigated the catalytic mechanism by SbzP. A steady-state kinetic assay suggested its catalytic mechanism to be the Ping-Pong Bi-Bi mechanism. Combining the isotope labeling experiment of deuterium-labeled SAM, stopped-flow photospectroscopy, and site-directed point mutatgenesis, they further proposed a reaction mechanism in which the active site lysine forms the β,γ-unsaturated quinonoid intermediate by a PLP and a SAM followed by the removal of the *S*-adenosyl moiety (Fig.1). This intermediate further reacts with an NAD^+^ (oxidized form of NAD) via a tandem alkylation at the Cα and Cγ positions to yield the 6,5-bicyclic scaffold. Although both alkylation positions have been reported in PLP-dependent enzymes (Chen M. *et al*, 2020; Du Y. L. and Ryan K. S., 2019; Faulkner J. R. *et al*, 2006; Hai Y. *et al*, 2020), SbzP can uniquely catalyze two alkylations to complete the [3+2]-cycloaddition reaction. Interestingly, SbzP is also phylogenetically distinct from other PLP enzymes, suggesting that SbzP-related enzymes form a new family of PLP-dependent cyclase. The precise reaction mechanism, though, *i.e.*, whether the reaction is step-wise or concerted, remains to be determined. Finally, by bioinformatic analysis of publicly available bacterial genomes, the authors identified SbzP homologs in multiple phyla, suggesting the wide distribution of NAD-utilizing specialized metabolism in bacteria.

The discovery of a NAD-utilizing biosynthetic enzyme is eye-opening, as generally cofactors are believed to function exclusively in assisting biochemical transformations during enzyme catalysis, but not being transformed itself. Abe, Awakawa, and co-workers nicely demonstrated the “greediness” of Nature to utilize limited building blocks from a cofactor pool to expand the structural variety of specialized metabolites. Biosynthetic pathways deviating from major biosynthetic classifications are often challenging for gene cluster discovery because there are no reliable retro-biosynthetic analyses possible. The resistance gene-guided genome mining (Yan Y. *et al*, 2020), as exemplified here by the altemicidin biosynthetic pathway discovery, can be a promising approach for disclosing such uncanonical and intriguing biosynthetic transformations.

Interestingly, recent studies have revealed the functional role of NAD as an ADP-ribosyl donor for protein and DNA modification (Cantó C. *et al*, 2015; Houtkooper R. H. *et al*, 2010; Schuller M. *et al*, 2021), illuminating another important function of enzyme cofactors in cell regulation. These reports together indicate that investigation of the sophisticated metabolism and biochemistry in living organisms have just begun, and nature is still filled with intriguing catalytic potential. We can envision that other cofactors, such as flavin adenine dinucleotide (FAD) and thiamine diphosphate (ThDP), can also potentially be utilized as building blocks in the specialized metabolism of microbes in nature that awaits discovery. It also raises interesting questions such as how microbes balance the metabolic flow of NAD between its primary role as a cofactor and its secondary role in the specialized metabolism that eventually leads to NAD consumption. Overall, the biosynthetic investigation of altemicidin described in this study adds a new page on the role of NAD cofactor and showcases an elegant biocatalytic transformation bridging primary metabolism and specialized metabolism.

## Declaration of competing interest

The authors declare that they have no known competing financial interests or personal relationships that could have appeared to influence the work reported in this paper.
